# Flux-Type versus Concentration-Type Sensors in Transdermal Measurements

**DOI:** 10.3390/bios13090845

**Published:** 2023-08-25

**Authors:** Bob M. Lansdorp

**Affiliations:** Milo Sensors, Inc., Santa Barbara, CA 93101, USA; bob@milosensor.com

**Keywords:** transdermal, biosensor, diffusion, flux, concentration, skin, timescale

## Abstract

New transdermal biosensors measure analytes that diffuse from the bloodstream through the skin, making it important to reduce the system response time and understand measurement output. While highly customized models have been created for specific sensors, a generalized model for transdermal sensor systems is lacking. Here, a simple one-dimensional diffusion model was used to characterize the measurement system and classify biosensors as either flux types or concentration types. Results showed that flux-type sensors have significantly faster response times than concentration sensors. Furthermore, flux sensors do not measure concentration, but rather have an output measurement that is proportional to skin permeability. These findings should lead to an improved understanding of transdermal measurements and their relation to blood analyte concentration. In the realm of alcohol research, where the majority of commercially available sensors are flux types, our work advocates toward moving away from transdermal alcohol concentration as a metric, and instead suggests embracing transdermal alcohol flux as a more suitable alternative.

## 1. Introduction

Non-invasive measurement of physiological health parameters has been a longstanding aspiration for scientists and engineers. Needle-based invasive glucose sensors, such as the Dexcom G6 and Abbott FreeStyle Libre 2, have made tremendous academic and commercial progress [1], and have helped millions of people to monitor their glucose levels. While microneedle-based sensors have been extensively discussed in academic literature [2], their regulatory challenges with the Food and Drug Administration (FDA), compounded by confusing FDA guidance on microneedling products [3], have hindered their practicality. Reverse iontophoresis, another topic of academic discourse [4], often overlooks the skin irritation issues experienced by the GlucoWatch Biographer and its users [5,6]. Consequently, invasive sensors relying on microneedles or iontophoresis face FDA regulation, presenting significant cost and time barriers for development beyond the academic prototype stage [7]. Optical transdermal sensing, pioneered by the optical oxygen sensor [8,9], has shown promising developments in sensing molecule concentrations through the skin [10]. However, challenges posed by the scattering of skin tissue have hindered the commercialization of this approach beyond oxygen measurement, which falls outside the scope of this study. Sweat-based sensors have also garnered attention [11], but ensuring active perspiration is not feasible for many applications outside of sports monitoring. Therefore, this study focuses on non-invasive biosensors that rely on the passive diffusion of molecules through the skin, known as transdermal insensible perspiration.

Numerous models have been proposed for the transdermal diffusion of analytes, primarily centered around delivering drugs into the bloodstream via Transdermal drug delivery (TDD) skin patches [12,13]. TDD research mainly focuses on the flux of molecules from the patch into the skin and bloodstream, whereas our study concentrates on the measurement of analytes present in the bloodstream using an external sensor. A diffusion model has been widely used in the modeling of skin permeation [14,15].

Ethanol presents an interesting case study for transdermal diffusion. However, confusion persists in the field regarding transdermal alcohol concentration and its relation to blood alcohol concentration, going back to early attempts to correlate the two [16]. Addressing the confusion surrounding transdermal flux, blood concentration, and their relationship to skin permeability constitutes a primary objective of this study. Several models have been proposed for ethanol diffusion, including a two-layer skin model [17] and a detailed analysis of the SCRAM sensor’s sensitivity to various experimental parameters [18]. While some early studies [17] provided valuable parameters, their intricate nature limits their applicability to other systems. Other studies on ethanol diffusion [19,20] excessively focus on mathematical methods for deconvoluting sensor responses, neglecting physical factors like skin permeability. Instead of fitting a particular skin–sensor system to a model, this study takes the perspective that a sensor’s rational design can be controlled to achieve an optimal response time. Although a few studies have attempted to quantify alcohol sensor outputs in physical units [21,22], many have omitted raw sensor measurement units [20,23,24,25,26], further contributing to the confusion regarding the relationship between Transdermal Alcohol Flux (TAF) and Blood Alcohol Concentration (BAC). The need to move away from a TAC-BAC equivalence has been qualitatively contemplated [24], and this article provides a quantitative framework for understanding the distinction.

The diffusion coefficient (*D*) and the effective thickness (*L*) of the skin are vital parameters, determined by human physiology, which influence the physics of transdermal molecular detection. While these parameters are beyond an experimenter’s control, the sensor’s sensitivity (α) can be manipulated. In this article, we explore extreme scenarios by setting α to zero and infinity, calculating the expected sensor response functions for the Neumann boundary condition when α is small and for the Dirichlet condition when α is large. The boundary condition choice, determined by the sensor type, significantly influences the expected response, as demonstrated in this study.

## 2. Methods

In order to reduce the complex problem of molecular diffusion into a mathematically tractable solution that provides meaningful guidance to engineers, we must make some simplifying assumptions. As described in the work by Mitragotri: “The ultimate challenge is probably to keep models simple enough, so that it is used by the experimental community, while still explaining some complex real world data” [13]. The goals of this study are to measure the influence of the sensor type (flux-type or concentration-type sensor) on the response time, as well as provide insight into the relation between skin permeability and sensor response. We focus on key variables, such as the sensor type and the diffusion coefficient of a given molecule through the skin.

**Figure 1:**
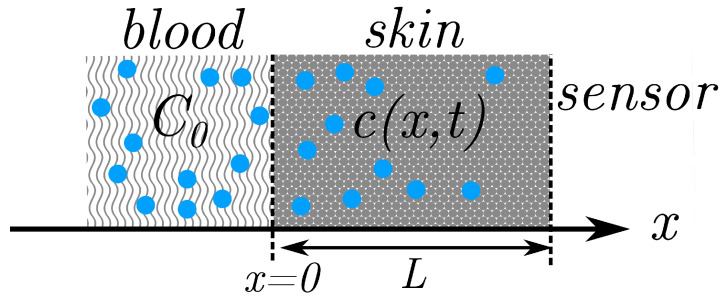
Schematic of one-dimensional geometry used to model diffusion of analyte molecules (blue dots) from the blood, through the skin, and into a sensor.

We assume a one-dimensional model along the x-direction, disregarding surface heterogeneity of the skin, with concentration that varies over both space and time c(x,t). The skin is modeled as a single layer with a thickness *L*, where the capillary bed is at x=0 and the skin/sensor interface is at x=L. We neglect analyte metabolism within the skin layers and assume unity solubility (partition coefficient) throughout. A schematic of the geometry is shown in Figure 1.

Next, we assume Fickian diffusion within the skin layer:

(1) $$\frac{\partial c(x,t)}{\partial t}=D\frac{\partial^{2}c\left(x,t\right)}{\partial x^{2}}$$

Our primary focus is to examine how the boundary condition at the skin/sensor surface influences the overall system performance. Here, we take three illustrative boundary conditions, defined as flux-type, concentration-type, and Robin-type conditions. In these definitions, a concentration-type sensor reversibly measures the molecules of interest and, therefore, consumes none of the molecules at the skin/sensor interface and does not allow any molecules to escape outward, such that we have a Neumann boundary condition with

(2) $${\left.\frac{\partial c}{dx}\right|}_{x=L}=0.$$

A flux-type sensor consumes all molecules at the skin/sensor interface by irreversibly catalyzing them and imposing a Dirichlet condition:

(3) c(L,t)=0.

A Robin-type sensor imposes a flux that is proportional to the concentration, using α, which is proportional to the electrode’s standard heterogeneous rate constant, as the constant of proportionality:

(4) $$D{\left.\frac{\partial c}{dx}\right|}_{x=L}=-\alpha c\left(L,t\right).$$

For all sensors, the initial condition is set as follows:

(5) c(x,0)=0.

At the blood–skin interface (x=0), an increase in concentration from zero to $C_{0}$ is imposed:

(6) $$c\left(0,t\right)=C_{0},t\geq 0.$$

### Sensors Types and Response Time

Here, we solve the diffusion equation and examine the expected response times for different representative sensor types: concentration-type, ideal flux-type, and Robin-type sensors. Concentration-type sensors measure the concentration of analytes in the sensor without allowing analyte molecules to pass through. Flux-type sensors measure the flux of analytes into the sensor. Ideal flux-type sensors irreversibly consume all the molecules at the sensor–skin interface. Robin-type sensors are flux sensors that impose a flux proportional to the concentration with the constant of proportionality α.

We use a separation of variables approach [27] on the relevant equations and boundary conditions (Equations (1)–(6)); we can obtain the measurement output versus time. We provide a reproducible code to solve the equations in Supplemental Mathematica Source Code, https://github.com/boblansdorp/diffusion-physics (accessed on 22 August 2023). We note that while the literature is clear about the Dirichlet and Neumann boundary condition solutions, the Robin boundary condition is left as a homework exercise in [27]. We solved it explicitly in Appendix A by following known methods [28].

For a concentration-type sensor with a Neumann boundary condition (Equation (2)), the concentration as a function of time and space, c(x,t), in response to a step change in concentration is given by

(7) $$c_{Conc}\left(x,t\right)=C_{0}\left(1-\frac{4}{\pi }\;\sum_{i=1}^{\infty }\left(\frac{1}{2i-1}\right)e^{\frac{-D\pi^{2}t{\left(2i-1\right)}^{2}}{4L^{2}}}\sin\frac{\pi x(2i-1)}{2L}\right)$$

The expected measurement response, m(t), for a concentration-type sensor is obtained by evaluating Equation (7) at the skin–sensor boundary:

(8) $$m_{Conc}\left(t\right)=c_{Conc}\left(L,t\right)=C_{0}\left(1+\frac{4}{\pi }\;\sum_{i=1}^{\infty }\left(\frac{1}{2i-1}\right)e^{\frac{-D\pi^{2}t{\left(2i-1\right)}^{2}}{4L^{2}}}\cos\pi i\right)$$

For an ideal flux-type sensor with a Dirichlet boundary condition (Equation (3)), the concentration as a function of time and space can be solved [29], and is given by

(9) $$c_{Flux}\left(x,t\right)=C_{0}\left(1-\frac{x}{L}-\frac{2}{\pi }\;\sum_{i=1}^{\infty }\left(\frac{1}{i}\right)e^{\frac{-D\pi^{2}ti^{2}}{L^{2}}}\sin\frac{\pi xi}{L}\right)$$

The measurement response for an ideal flux-type sensor $m_{Flux}\left(t\right)$ scales with the flux of molecules into the sensor:

(10) $$m_{Flux}\left(t\right)\propto -D\frac{\partial c(x,t)}{\partial x}=\frac{C_{0}D}{L}\left(1+2\sum_{i=1}^{\infty }e^{\frac{-D\pi^{2}ti^{2}}{L^{2}}}\cos\pi i\right)$$

As shown in Appendix A, the concentration at the skin/sensor interface for a Robin boundary condition (Equation (4)), as a function of time, is

(11) $$c_{Robin}\left(x,t\right)=C_{0}\left(\sum_{n=1}^{\infty }b_{n}\sin\left(\frac{\mu_{n}x}{L}\right)e^{\frac{-\mu_{n}^{2}tD}{L^{2}}}+1-\frac{\frac{\alpha L}{D}\frac{x}{L}}{1+\frac{\alpha L}{D}}\right)$$

and the sensor response is

(12) $$m_{Robin}\left(t\right)=\alpha C_{0}\left(\sum_{n=1}^{\infty }b_{n}\sin\left(\mu_{n}\right)e^{\frac{-\mu_{n}^{2}tD}{L^{2}}}+\frac{1}{1+\frac{\alpha L}{D}}\right)$$

where the coefficients $\mu_{n}$ can be found numerically (see Appendix A).

## 3. Results

Here, we plot the spatial and temporal results of the diffusion equation solutions over the skin and then examine the temporal responses. The Mathematica source code used to generate these figures can be found at https://github.com/boblansdorp/diffusion-physics (accessed on 22 August 2023).

### 3.1. Spatial Results

**Figure 2:**
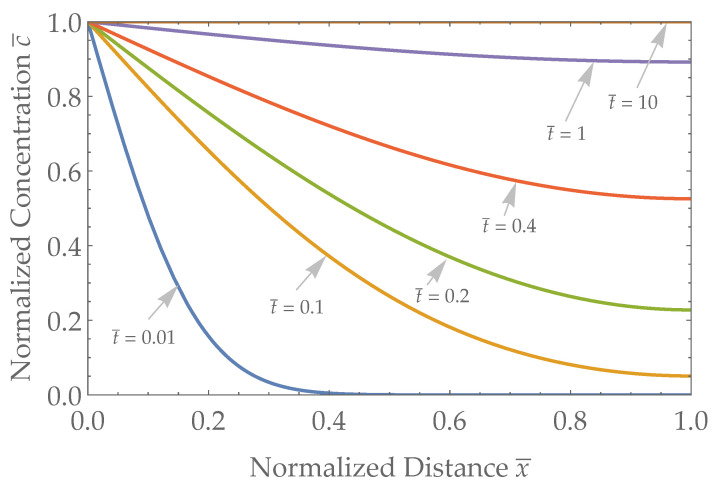
The concentration of a given analyte as a function of space for a few representative times with a Neumann boundary condition at the skin–sensor interface. Distance ranges from $\bar{x}=0$ (blood/skin interface) to $\bar{x}=1$ (skin/sensor interface) and representative times range from $\bar{t}=0.01$ to $\bar{t}=10$ after a step change in concentration at $\bar{x}=0$.

The results of the concentration versus space and time for a Neumann boundary condition, using Equation (7), are plotted in Figure 2. Time is nondimensionalized as $\bar{t}=tD/L^{2}$, distance within the skin interface is nondimensionalized as $\bar{x}=x/L$, and concentration is nondimensionalized as $\bar{c}=c/C_{0}$, so as to make the results as universally applicable as possible. As time progresses, the concentration at the skin/sensor boundary ($\bar{x}=1$) gradually reaches the blood concentration ($c=C_{0}$, or equivalently, $\bar{c}=1)$.

**Figure 3:**
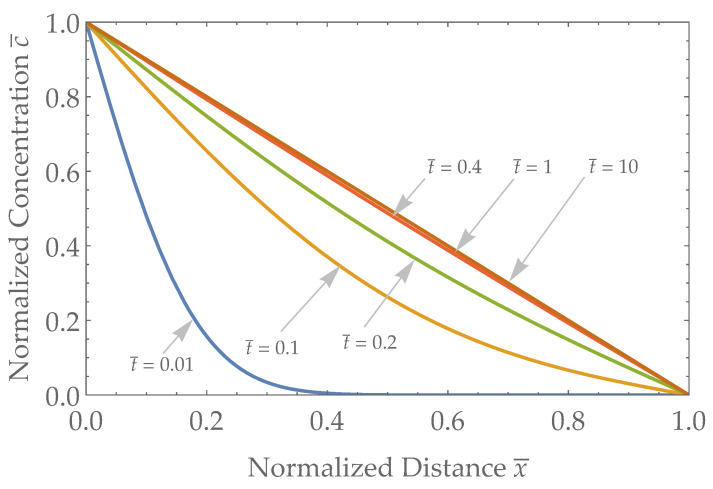
The concentration of a given analyte as a function of space for a few representative times with a Dirichlet boundary condition at the skin–sensor interface. Distance ranges from $\bar{x}=0$ (blood/skin interface) to $\bar{x}=1$ (skin/sensor interface) and representative times range from $\bar{t}=0.01$ to $\bar{t}=10$ after a step change in concentration at $\bar{x}=0$.

The ideal flux-type sensor boundary condition results from Equation (9) are plotted in Figure 3, with time, space, and concentration nondimensionalized as above.

**Figure 4:**
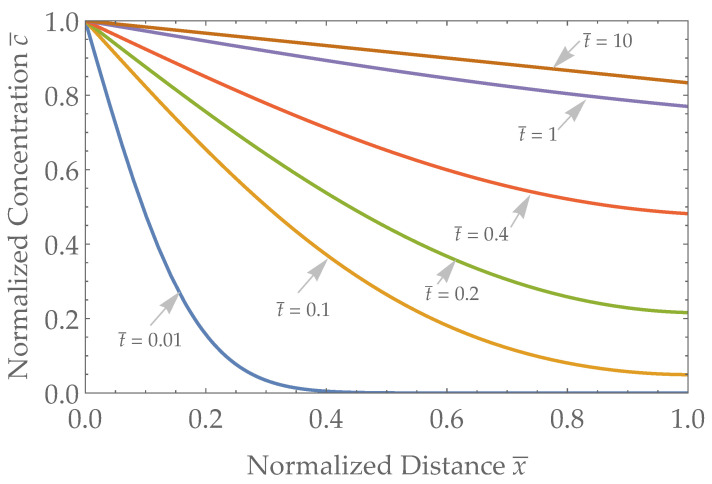
Analyte Concentration versus distance in the skin layer for a Robin-type sensor with α=D/L. Distance ranges from $\bar{x}=0$ (blood/skin interface) to $\bar{x}=1$ (skin/sensor interface) and representative times range from $\bar{t}=0.01$ to $\bar{t}=10$ after a step change in concentration at $\bar{x}=0$.

The Robin boundary equation solution (Equation (11)) with a representative intermediate value of α=D/L is used to generate the results in Figure 4.

### 3.2. Sensor Response Times

Concentration- and flux-type sensors have different units for their measurement outputs (see Equations (7) and (9)). Concentration sensors measure concentration $\mathrm{mol}{\mathrm{cm}}^{-3}$ whereas flux-type and Robin-type sensors measure flux in $\mathrm{mol}{\mathrm{cm}}^{-2}s^{-1}$. To be able to directly compare the time responses of concentration sensors versus flux-type sensors, we non-dimensionalize their outputs by their steady-state values. The steady-state response of a concentration-type sensor is

$$m_{Conc,Steady-State}=\lim_{t\to \infty }c_{Conc}\left(L,t\right)=C_{0},$$

and the nondimensionalized sensor response for a concentration-type sensor is

(13) $${\bar{m}}_{Conc}=\frac{m_{Conc}\left(t\right)}{m_{Conc,Steady-State}}=1+\frac{4}{\pi }\;\sum_{i=1}^{\infty }\left(\frac{1}{2i-1}\right)e^{\frac{-D\pi^{2}t{\left(2i-1\right)}^{2}}{4L^{2}}}\cos\pi i.$$

Similarly, a flux sensor has

(14) $$m_{Flux,Steady-State}=C_{0}\frac{D}{L},$$

whereby the nondimensionalized sensor response is

(15) $${\bar{m}}_{Flux}=\frac{m_{Flux}\left(t\right)}{m_{Flux,Steady-State}}=1+2\sum_{i=1}^{\infty }e^{\frac{-D\pi^{2}ti^{2}}{L^{2}}}\cos\pi i.$$

The steady-state magnitude of the signal of a Robin sensor is

(16) $$m_{Robin,Steady-State}=C_{0}\alpha \frac{1}{1+\frac{\alpha L}{D}},$$

and taking $m_{Robin}\left(t\right)$ from the appendix, Equation (A26), we obtain the following:

(17) $${\bar{m}}_{Robin}=\frac{m_{Robin}\left(t\right)}{m_{Robin,Steady-State}}=1+\left(\left(1+\frac{\alpha L}{D}\right)\sum_{n=1}^{\infty }b_{n}\sin\left(\mu_{n}\right)e^{\frac{-\mu_{n}^{2}tD}{L^{2}}}\right).$$

**Figure 5:**
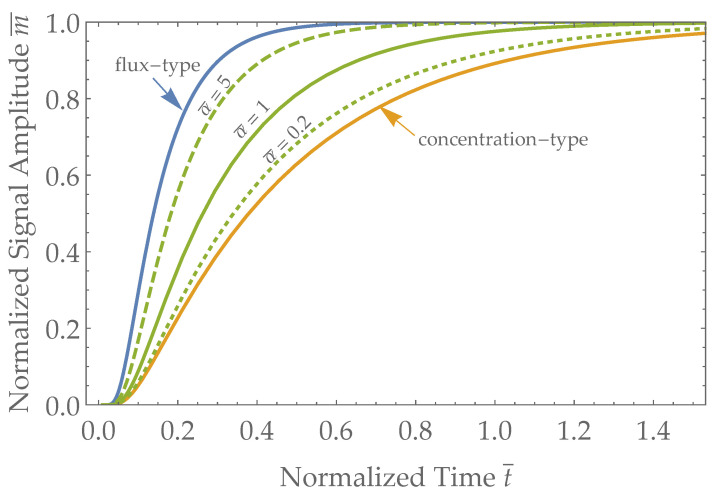
The expected normalized sensor response versus time for a step change in concentration for a concentration-type sensor with a Neumann boundary condition (${\bar{m}}_{Conc}$, Equation (13)—orange), an ideal flux-type sensor with a Dirichlet boundary condition (${\bar{m}}_{Flux}$, Equation (15)—blue); intermediate values are represented by three Robin-type sensors (${\bar{m}}_{Robin}$, Equation (17)—green). The only free parameter α, which influences the Robin-type sensor response, is varied, as follows: α=D/(5L) (dotted), α=D/L (solid), and α=5D/L (dashed). Time is nondimensionalized as $\bar{t}=tD/L^{2}$ to allow any combination of *D* and *L* to be readily compared across different sensor types. We note that the combined skin-plus-sensor system becomes faster as the sensor sensitivity α increases with respect to skin permeability D/L (i.e., increasing $\bar{\alpha }$ green curves), up to the limit of the response time dictated by a flux-type sensor (blue curve).

We can further non-dimensionalize the system by considering the sensor rate constant α in relation to skin permeability $k_{\mathrm{Skin}}=D/L$, and defining $\bar{\alpha }=\alpha /k_{\mathrm{Skin}}=\frac{\alpha L}{D}$. In Figure 5, we plot the nondimensionalized sensor responses for a concentration-type sensor ${\bar{m}}_{Conc}$, an ideal flux-type sensor ${\bar{m}}_{Flux}$, and Robin-type sensors ${\bar{m}}_{Robin}$ (with α set to representative values of α=D/(5L), α=D/L, and α=5D/L, equivalent to $\bar{\alpha }=0.2$, $\bar{\alpha }=1$, and $\bar{\alpha }=5$, respectively).

## 4. Discussion

In a mathematical sense, this entire paper could be reduced to a relatively trivial discussion of Dirichlet versus Neumann boundary conditions on a second-order linear differential equation. However, in the field of transdermal biosensors, simplification and reduction to basic principles are much needed! Whereas the sensor is often thought of as independent from the measurement system, here, we show that the sensor type choice can dramatically impact the type of response that is expected. In this paper, we examine the influence of sensor sensitivity α in relation to the skin permeability $k_{\mathrm{Skin}}=D/L$.

### 4.1. Robin Sensor in Extreme Limits

The Robin boundary condition (Equation (4) can be examined in two limits: α≫D/L and α≪D/L.

Rewriting it as $\lim_{\frac{\alpha L}{D}\to \infty }\frac{D}{\alpha }{\left.\frac{\partial c}{dx}\right|}_{x=L}=-c\left(L,t\right)$, we can see that the Robin condition becomes equal to the Dirichlet condition c(L,t)=0 in the case of the sensor with sensitivity that is much greater than skin permeability. Thus, a system that includes a sensor with an effective sensitivity (also known as the rate constant), which is much greater than skin permeability, can be approximated as an ideal flux-type sensor system.

Similarly, when $\lim_{\frac{\alpha }{D/L}\to 0}D{\left.\frac{\partial c}{dx}\right|}_{x=L}=-\alpha c\left(L,t\right)$, it yields $D{\left.\frac{\partial c}{dx}\right|}_{x=L}=0$, which is the Neumann boundary condition. Thus, very low-sensitivity sensors effectively result in a system that can be approximated as a concentration-type time response system with a flux-type amplitude.

### 4.2. Sensor Types and Expected Response Times

Upon examination of Figure 5, it is clear that the ideal flux-type sensor is faster than a concentration sensor responding to changes in the blood analyte concentration. Furthermore, the Robin boundary condition represents an intermediate result in terms of sensor response timescales between a concentration-type sensor and an ideal flux-type sensor. It can be helpful to think about timescales in terms of the extreme cases of either ideal flux-type or concentration-type sensors to establish the bounds for the timescales of response in actual Robin-type sensors.

Having calculated the expected sensor response to a concentration step change for both flux-type and concentration-type sensors, a natural question is ’how much faster are flux-type sensors than concentration sensors, all else being equal?’ Comparing Equations (10) and (8), we can see that flux-type and concentration-type transdermal sensors have different functional forms, which means that one is not simply a constant faster than the other. There are an infinite number of possible metrics used to quantify the difference, but two logical ones that have been used in the past include examining the exponent of the leading term in the series, and integrating the sensor response, taking the x-intercept [29]. Examining Equation (10), we find the leading term (i=1) to have in the exponent a value of $\frac{D\pi^{2}t}{L^{2}}$, giving us a timescale of

(18) $$\tau_{Flux}=\frac{L^{2}}{D\pi^{2}}.$$

Comparing that to the leading term of Equation (8) of

(19) $$\tau_{Conc}=\frac{4L^{2}}{D\pi^{2}}\;$$

we find that the ratio of timescales is $\tau_{Conc}/\tau_{Flux}=4$. Thus, for individual-specific *D* and *L*, an ideal Flux-type sensor is four times faster than a concentration-type sensor. Similarly, using the integrated method, we find that the integrated measurement signal converges on a line $\int_{0}^{\infty }{\bar{m}}_{Flux}\left(t\right)dt=\left(t-t_{Flux}\right)$ and the concentration sensor converges on $\int_{0}^{\infty }{\bar{m}}_{Conc}dt=\left(t-t_{Conc}\right)$, from which we find the ratio $t_{Conc}/t_{Flux}$ numerically (see Supplemental Mathematica Source Code, https://github.com/boblansdorp/diffusion-physics (accessed on 22 August 2023)) to be equal to approximately 3. Depending on the method of comparison that is chosen, the exact number can vary, but what is clear is that for a fixed *D* and *L* given by an individual’s physiology, ideal flux-type sensors are approximately three or four times faster than concentration-type sensors.

### 4.3. Amplitude of Flux Type Sensors

Concentration-type sensors intuitively measure in units of concentration ($\mathrm{mol}{\mathrm{cm}}^{-3}$), whereas flux-type sensors signal in units of $\mathrm{mol}{\mathrm{cm}}^{-2}s^{-1}$, and have a pre-factor of D/L that relates the measured sensor signal $m_{Flux}$ to the blood analyte concentration $C_{0}$. The parameter, D/L, is often called the permeability $k_{\mathrm{Skin}}$, which has units of $\mathrm{cm}s^{-1}$. In the case of a transdermal biosensor, the permeability $k_{\mathrm{Skin}}$ is a function of many parameters, including skin thickness and hydration, and is highly molecule-specific. Furthermore, skin permeability will typically vary as a function of time as an individual increases their temperature, hydration, or any number of factors. Thus, unless permeability can be estimated in real time, flux-type transdermal biosensors will never accurately report blood analyte concentrations.

### 4.4. Alcohol Sensors as a Case Study

As a representative case study of transdermal diffusion, we can take ethanol (the molecule present in alcoholic beverages that causes intoxication). We use previously published estimates [30] for the diffusion coefficient of ethanol in the skin of approximately $D=3\times 10^{-11}{\mathrm{cm}}^{2}s^{-1}$ and the rate-limiting layer of skin (stratum corneum) with a thickness of L=15 μm [17]. We use the values for the dry stratum corneum, not for the fully hydrated layer, and assume a consistent solubility. We can obtain a relevant timescale using the leading terms of Equations (18) and (19) as $\tau_{Conc}\approx \frac{4L^{2}}{\pi^{2}D}=8.4\;h$ and $\tau_{Flux}\approx \frac{L^{2}}{\pi^{2}D}=2.1\;h$. Thus, we can expect transdermal alcohol sensors to have response times between 2.1 h and 8.4 h, depending on the sensor sensitivity. These timescales are in agreement with previous experimentally measured results for transdermal timescales [31].

We can also calculate $\bar{\alpha }$ to obtain the relative sensor rate constant in relation to skin permeability $k_{\mathrm{Skin}}$. For an enzymatic sensor with a diffusion-limiting membrane [32]:

$$\alpha_{Enzymatic}=1.3\times 10^{-7}\mathrm{cm}s^{-1}.$$

We can compare this to the mass transfer coefficient of human skin for ethanol, given by $k_{\mathrm{Skin}}=\frac{D}{L}=\frac{5\times 10^{-10}\;{\mathrm{cm}}^{2}s^{-1}}{15\;\mu m}=3.3\times 10^{-7}\mathrm{cm}s^{-1}$; thus, $\bar{\alpha }=\frac{\alpha_{Sensor}}{k_{Skin}}=0.4$, which is less than one. Thus, the enzymatic wearable alcohol sensor (e.g., ION RAP) with a diffusion-limiting membrane is a Robin-type sensor with $\bar{\alpha }=0.4$, which will exhibit a temporal response of a concentration-type sensor and the measurement output of a flux-type sensor (whose amplitude is sensitive to skin permeability).

For the same enzymatic sensor with no diffusion-limiting membrane, for example, with an enzyme-limited sensitivity of 200 nA at an ethanol concentration of 200 μ$\mathrm{mol}L^{-1}$ (data not published), with a surface area of $A=0.5\;{\mathrm{cm}}^{2}$, Faraday’s constant of $F=9.64\times 10^{4}\;sA{\mathrm{mol}}^{-1}$, and assuming n=2 electrons per ethanol molecule, we can deduce the sensor’s mass transport coefficient:

$$\alpha_{Enzymatic}=\frac{i}{CnAF}=\frac{200\times 10^{-9}\;A\times 10^{3}\;{\mathrm{cm}}^{3}L^{-1}}{200\;\mu \mathrm{mol}L^{-1}\times 2\times 0.5\;{\mathrm{cm}}^{2}\times 9.64\times 10^{4}\;sA{\mathrm{mol}}^{-1}}=1.0\times 10^{-5}\;\mathrm{cm}s^{-1}$$

Here, the non-dimensionalized sensor sensitivity is $\bar{\alpha }=\frac{\alpha_{Sensor}}{k_{Skin}}=31$, which is much greater than one. Thus, the enzymatic wearable alcohol sensor without a diffusion-limiting membrane (e.g., ION Wearable) can be well-approximated as an ideal flux-type sensor.

We can perform a similar analysis for WrisTAS [33] with a diffusion-limiting membrane. With $1\;mg{\mathrm{dL}}^{-1}$ ethanol having a molecular weight of $46.07\;g{\mathrm{mol}}^{-1}$, we obtain the following:

$$\alpha_{WrisTAS}=\frac{i}{CnAF}=\frac{8\times 10^{-6}\;A\times 1\times 10^{2}\;{\mathrm{cm}}^{3}{\mathrm{dL}}^{-1}\times 46.07\;g{\mathrm{mol}}^{-1}}{100\;mg{\mathrm{dL}}^{-1}\times 1\times 10^{-3}\;g{\mathrm{mg}}^{-1}\times 4\times 3.88\;{\mathrm{cm}}^{2}\times 9.64\times 10^{4}\;sA{\mathrm{mol}}^{-1}}$$

$$\alpha_{WrisTAS}=2.45\times 10^{-7}\;\mathrm{cm}s^{-1}$$

This yields a ratio of the sensor activity to skin permeability, as $\bar{\alpha }=\frac{\alpha_{Sensor}}{k_{Skin}}=\frac{2.45\times 10^{-7}\;\mathrm{cm}s^{-1}}{3.3\times 10^{-7}\;\mathrm{cm}s^{-1}}=0.75$, which is approximately one. Thus, WrisTAS can neither be approximated as an ideal flux-type sensor nor as a concentration sensor, but is a Robin-type sensor with $\bar{\alpha }=0.75$. We note that the calculations in the patent [33] assume a flux of $1.15\times 10^{-11}\;\mathrm{mol}{\mathrm{cm}}^{-2}s^{-1}$ at a concentration of $100\;mg{\mathrm{dL}}^{-1}$, from which we can deduce

$$k_{skin,WrisTASpatent}=\frac{1.15\times 10^{-11}\;\mathrm{mol}{\mathrm{cm}}^{-2}s^{-1}\times 46.07\;g{\mathrm{mol}}^{-1}\times 100\;{\mathrm{cm}}^{3}{\mathrm{dL}}^{-1}}{100\;mg{\mathrm{dL}}^{-1}\times 1\times 10^{-3}\;g{\mathrm{mg}}^{-1}}$$

$$=5.3\times 10^{-7}\;\mathrm{cm}s^{-1},$$

which differs slightly from the $3.3\times 10^{-7}\;\mathrm{cm}s^{-1}$ used in this manuscript, although both values were derived from the same 1971 Scheuplein article [30]. Data on skin permeability to ethanol, especially in real-world conditions and across a diverse population, are scarce.

If the raw sensor data were provided, we could perform similar calculations for BACTrack Skyn, the SCRAM ankle bracelet, and other products, and could ask “is this sensor more flux-type or more concentration-type in the presence of human skin?” Unfortunately, Skyn data provided to date have been in units of counts [23], not actual measurement units of μA. Publications that examine SCRAM [34] also show the output in units of concentration, presumably based on a laboratory calibration using a standard gas concentration, which makes an estimation of $\bar{\alpha }$ impossible. Anderson et al. have attempted to model SCRAM with a continuous airflow of $\dot{V}$ over the skin into a gas headspace with length $L_{g}$ [18]. Although the model is highly detailed, the applicability of this model to reality is questionable, since the SCRAM device they attempt to model pumps air every 30 min, whereas the authors assume a continuous flow rate of air. If SCRAM has a very high flow rate in the pump, it could be considered a flux-type sensor. In contrast, if the flow rate is low, it could be considered a Robin-type sensor with a low α, such that it has a timescale of a concentration sensor. If the pump samples at thirty-minute intervals with a minimal sampling volume, and integrates the the current over the duration derived from that volume (similar to how a breathalyzer functions), it could be considered a concentration-type sensor. An accurate measurement of the SCRAM pump flow rate, denoted as $\dot{V}$, is critical to understand its position on the spectrum between concentration- and flux-type sensors.

#### Recommendations for Alcohol Research

In the field of alcohol research, Blood Alcohol Concentration (BAC) is the gold standard metric for assessing intoxication levels. The term Transdermal Alcohol Concentration (TAC) may appear appealing as it implies some degree of equivalence. However, its use in the context of flux-type sensors, such as WrisTAS/BI Transdermal Alcohol Detector (TAD), BACtrack Skyn, ION Wearable, and (potentially) SCRAM CAM (depending on the volumetric flow rate), is misleading. For ideal flux-type sensors, TAC remains consistently zero over time, even though BAC and TAF exhibit temporal variability (see $\bar{x}=1$ in Figure 3). Therefore, it is strongly recommended to adopt TAF as the output metric for flux-type sensors.

Equating TAF to BAC, or establishing correlations between the two without accounting for the pre-factor of skin permeability, is scientifically unsound, given their different units of measurement ($\mathrm{mol}{\mathrm{cm}}^{-2}s^{-1}$ and $\mathrm{mol}{\mathrm{cm}}^{-3}$, respectively). Researchers aiming to estimate BAC using flux-type alcohol sensors for transdermal measurements [21,23,25,26,35,36,37,38,39,40,41] need to consider several key aspects. These include preserving the raw sensor data (e.g., microamperes for flux-type platinum fuel cells or flux in $\mathrm{mol}s^{-1}{\mathrm{cm}}^{-2}$), determining the sensor sensitivity (e.g., conversion factor between microamperes and $\mathrm{mol}{\mathrm{dL}}^{-1}$ in controlled conditions [32]), and employing a comprehensive model for the skin and sensor system. The model should incorporate the time-dependent variability of skin permeability ($k_{\mathrm{Skin}}\left(t\right)$ in units of $\mathrm{cm}s^{-1}$). Neglecting these steps when attempting to correlate TAF with BAC disregards the fact that steady-state TAF for a flux-type sensor is the product of two components: BAC and skin permeability (see Equation (14)). The scatter that has been observed in TAF:BAC correlations can likely be attributed to variations in skin permeability. While assuming a model for skin permeability [32] may serve as a reasonable first approximation, it is essential to measure the variability of permeability over time and between individuals independently.

Although estimating BAC from TAF poses challenges without independent knowledge of skin permeability, researchers with access to TAF and concurrent BAC data, and who have an understanding of the sensor response function (e.g., $\alpha \gg k_{\mathrm{Skin}}$), can make estimates on skin permeability (in units of $\mathrm{cm}s^{-1}$) and variations across individuals in real-world environments. Existing research is nearing the accomplishment of this objective [19,20,42,43]. By retaining the raw sensor units (e.g., microamperes) and employing a model to determine the sensor response (e.g., the number of liberated electrons per ethanol molecule) to derive flux in $\mathrm{mol}{\mathrm{cm}}^{-2}s^{-1}$, it should be possible to combine this information with existing diffusion models to estimate skin permeability in $\mathrm{cm}s^{-1}$ across individuals and over time. Introducing some constraints on real-world ethanol permeability through the stratum corneum would be a valuable addition to the expanding body of research in this field.

Alternatively, it would be highly beneficial if a researcher could develop a novel measurement technique to directly assess skin permeability to ethanol alongside TAF measurements. This combination of metrics could lead to a more accurate estimation of BAC compared to relying on TAF alone.

### 4.5. Examples of Sensor Types and Applicability

The main objective of this paper is to present a comparison of flux-type (Dirichlet) sensors as compared to concentration-type (Neumann) sensors. Examples of flux-type sensors that irreversibly consume analytes during the detection process include amperometric sensors, such as platinum fuel cells [44], platinum electrodes [26], or enzymatic sensors [32]. Examples of concentration-type sensors that reversibly interact with analyte molecules include fiber-optic probes [45], molecularly imprinted polymers [46], potentiometric sensors [47], solution-gated transistor sensors [48], and aptamer-based sensors [49]. Antigen–antibody binding systems represent an interesting intermediate where binding is theoretically reversible, but may be kinetically limited so as to be effectively irreversible in practical measurement timescales [50].

Molecules that may be candidates for transdermal detection with relevance to this work include water [14,15], ethanol [15,16,17,18,19,20,21,23,24,31,34,39], glucose [51], nicotinamide [15], testosterone [15], caffeine [52], vitamin D [53], drugs of abuse, such as cocaine, codeine, and heroin [54,55], pharmaceutical drugs, such as aspirin, ibuprofen, and naproxen [56], nicotine [57], acetaldehyde [58], methanol [58], other volatile organic compounds [59], and potentially many more. It is worth noting that these molecules may have diffusion coefficients that vary by three orders of magnitude or more, resulting in transdermal response times that may vary from minutes to days, depending on the molecule.

### 4.6. Sensor Design Guide

When it comes to designing a sensor, the choice between a concentration-type sensor and a flux-type sensor has implications for both the response time and readout amplitude. Accurate knowledge of concentration is crucial in numerous medical applications, and a fast response time is essential for establishing a meaningful connection between measured signals and blood analyte concentrations. A fast response time is particularly important in scenarios where real-time interventions based on sensor readings are desired. A concentration-type sensor provides readings in familiar concentration units, but its response time is slower. On the other hand, a flux-type sensor offers faster response times, but its output is proportional to the skin permeability of the target molecule.

When designing a flux-type sensor, it is crucial to ensure that its sensitivity is sufficiently high, with the mass-transfer coefficient (in cm/s) exceeding the skin permeability ($\alpha_{\mathrm{sensor}}\gg k_{\mathrm{skin}}=D/L$). This prevents unnecessary delays in the system response time. However, increasing sensitivity too far may also lead to increased noise, resulting in an engineering trade-off between the response time and signal-to-noise ratio.

For instance, consider a transdermal alcohol sensor system. If a concentration-type sensor has a response time of $\tau_{\mathrm{Conc}}\approx 8.4\;h$, while the physiological alcohol concentration can change significantly within $\tau_{\mathrm{Physiology}}\approx 0.5\;h$, the concentration-type sensor will struggle to accurately estimate BAC. Similarly, a flux-type sensor with $\tau_{\mathrm{Flux}}\approx 2.1\;h$ will face challenges in estimating BAC accurately, as $\tau_{\mathrm{Flux}}>\tau_{\mathrm{Physiology}}$, and incomplete information on skin permeability ($k_{\mathrm{Skin}}$) further complicates the estimation. However, in scenarios where alcohol concentration is nominally zero, the flux-type sensor will be faster than the concentration sensor in detecting temporary excursions above zero.

### 4.7. Future Work and Recommendations

One avenue for future work could be to include more nuanced sensor responses. In Equations (8) and (10), we ignore the headspace that is often present between the skin and the sensor. Incorporating another layer here could be a logical next step. In this work, we ignore the brick-and-mortar-like structure of the stratum corneum, which has both hydrophilic and lipophilic components [60]. We also ignore shunt pathways, such as sweat glands. Future work could allow molecules to be either bound or unbound, as a method for modeling the diverse physiochemical environment of the stratum corneum.

This work draws from Figure 5 to conclude that flux-type sensors have a faster response than concentration-type transdermal sensors. A more rigorous derivation, for example, mathematically proving that ${\bar{m}}_{Flux}\left(t\right)$ is always greater than ${\bar{m}}_{Conc}\left(t\right)$, would be welcome.

Another potential avenue of exploration would be to extend the model to other molecules beyond ethanol. Mitragotri [13] describes how molecular free volume [61] can be used to predict effective diffusion versus molecular weight. The partition coefficient (variable solubility) was ignored in this manuscript, but it can be identified as a function of molecular weight in an analogous way [13]. The Potts–Guy Equation, in its commonly used form, is often referred to as a QSPR model, to describe the diffusion coefficient in the stratum corneum [13]. However, more advanced models would need to be constrained by real measurements of solubility and diffusion through live stratum corneum tissue; such measurements are currently lacking in the field.

It would be highly valuable to conduct measurements of skin permeability on a variety of molecules across diverse populations, in real-world environments, and across different regions of the human body. Such data would provide valuable insight for future research endeavors.

In this work, we derived the unit step response for different skin-plus-sensor systems. It is known that the unit impulse response is the derivative of the unit step response and that the unit impulse response can be used in linear invariant systems to perform a convolution integral and obtain expected results when there are time-varying boundary conditions. Extending the unit step responses derived in this work to further derive the unit impulse response would be an obvious next step. Additionally, performing a convolution integral, for example, to derive the expected TAF, given a time-varying BAC, could be a subsequent step. Then, experimental sensor data could be compared to independent physiological measurements to derive the best-fit unit impulse response. It would be of interest to measure how the unit impulse response and associated model parameters, such as skin thickness *L* and diffusion coefficient *D*, vary among different populations and environmental conditions. Once the reliability of the unit impulse response is established, deconvolution of measured signals, such as flux, into physiologically relevant signals, such as blood concentration, could be performed.

## 5. Conclusions

We demonstrated that flux-type sensors are significantly faster (three to four times) than concentration-type sensors when measuring step changes in blood analyte concentrations transdermally. However, it is important to note that flux-type sensors rely on skin permeability as a constant factor, relating the measured flux to the physiological concentration. Inaccurate knowledge of skin permeability can introduce uncertainty in estimating blood concentration accurately.

When designing a sensor, it is crucial to make an early decision on whether to pursue a concentration-type sensor or flux-type sensor for a specific application. For concentration-type sensors, designers should be aware that they will have relatively slow response times and should consider the timescales of the sensor–skin system in relation to physiologically relevant timescales. On the other hand, designers of flux-type sensors should aim to achieve sensitivity greater than the skin permeability and strive to acquire knowledge of skin permeability to establish a relationship between flux measurements and physiological concentrations.

In situations where the goal is to detect excursions above zero, a flux-type sensor will be faster and, therefore, more effective in accomplishing that objective.

## Data Availability

The Mathematica source code used to solve equations and generate figures is provided at https://github.com/boblansdorp/diffusion-physics (accessed on 22 August 2023).

## Abbreviations

**FDA**	Food and Drug Administration
**TDD**	Transdermal drug delivery
**BAC**	Blood Alcohol Concentration
**TAC**	Transdermal Alcohol Concentration
**TAF**	Transdermal Alcohol Flux
**TAD**	Transdermal Alcohol Detector

## Appendix A

Here, we derive an analytical solution to concentration *c* by using a Fickian diffusion equation with a Robin boundary condition at the skin/sensor interface. First, we transform the equations for *c* into a non-dimensional form $\bar{c}$ to make the equations easier to read during derivations. Then, we obtain a steady-state solution by assuming no time dependence. Then, we introduce a new variable $\hat{c}$ that homogenizes the boundary conditions. The result for $\hat{c}$ can be solved and converted back into $\bar{c}$ and *c*. Finally, we write the full analytical form of the diffusion equation with a Robin boundary condition.

The Robin boundary condition is

(A1) $$D{\left.\frac{\partial c(x,t)}{\partial x}\right|}_{x=L}=-\alpha c\left(L,t\right)$$

### Appendix A.1. Transformation of Variables

To make the following derivations easier to follow, we introduce non-dimensionalized versions of the variables *x*, *t*, c(x,t), which we denote as $\bar{x}$, $\bar{t}$, and $\bar{c}\left(\bar{x},\bar{t}\right)$, respectively, where

(A2) $$\bar{x}\equiv x/L$$

(A3) $$\bar{t}\equiv \frac{D}{L^{2}}t$$

(A4) $$\bar{\alpha }\equiv \frac{\alpha L}{D}$$

where

(A5) $$\bar{c}\left(\bar{x},\bar{t}\right)\equiv c(L\bar{x},L^{2}\bar{t}/D)/C_{0}$$

so that the diffusion equation (Equation (1)), boundary conditions, and initial condition can be rewritten as follows:

(A6) $$\frac{\partial^{2}\bar{c}}{\partial {\bar{x}}^{2}}=\frac{\partial \bar{c}}{\partial \bar{t}}$$

At the blood–skin interface ($\bar{x}=0$), we assume

(A7) $$\bar{c}\left(0,\bar{t}\right)=1,\bar{t}>0$$

At the skin–sensor interface (x=L, $\bar{x}=1$), we assume with a Robin boundary condition such that

(A8) $${\left.\frac{\partial \bar{c}}{\partial \bar{x}}\right|}_{\bar{x}=1}=-\bar{\alpha }\bar{c}\left(1,\bar{t}\right)$$

The initial condition can be rewritten as follows:

(A9) $$\bar{c}\left(\bar{x},0\right)=0$$

### Appendix A.2. Steady-State Solution

We define the steady-state solution as one that does not vary with time, e.g.,

$$\frac{\partial \bar{c}}{\partial \bar{t}}=0$$

and so then $\bar{c}=\bar{X}\left(\bar{x}\right)$, and with (A6), we obtain

$${\bar{X}}^{\prime \prime }=0$$

which yields the linear steady-state solution of

$$\bar{X}\left(\bar{x}\right)=A\bar{x}+B$$

with boundary conditions of $\bar{X}\left(0\right)=1=A\left(0\right)+B=B$.

The other boundary condition (A8) yields

(A10) $${\left.\frac{\partial \bar{c}\left(\bar{x},\bar{t}\right)}{\partial \bar{x}}\right|}_{\bar{x}=1}=A=-\bar{\alpha }\bar{c}\left(1,\bar{t}\right)=-\bar{\alpha }\left(A+B\right)$$

After manipulation, we obtain ${\bar{X}}_{0}=1-\frac{\bar{\alpha }x}{1+\bar{\alpha }}$ as the steady-state solution. We will use this to homogenize the boundary conditions. We call this solution the *steady-state solution* rather than the *equilibrium solution* because the system is not in thermodynamic equilibrium, since there is still a steady-state flux of material from the blood through the skin in this state.

### Appendix A.3. Homogenizing Boundary Conditions

Proceeding with the separation of variables at this point leads to difficulty since the boundary conditions are not homogeneous (e.g., they are not equal to zero). To homogenize the boundary conditions, we introduce another parameter:

(A11) $$\hat{c}=\bar{c}-{\bar{X}}_{0}$$

### Appendix A.4. Non-Dimensional Equations

We can rewrite the main diffusion condition to be

$$\frac{\partial^{2}\left(\hat{c}\left(\bar{x},\bar{t}\right)+{\bar{X}}_{0}\right)}{\partial {\bar{x}}^{2}}=\frac{\partial \left(\hat{c}\left(\bar{x},\bar{t}\right)+{\bar{X}}_{0}\right)}{\partial \bar{t}}$$

and since ${\bar{X}}_{0}$ is linear with *x*, we simply obtain

(A12) $$\frac{\partial^{2}\hat{c}\left(\bar{x},\bar{t}\right)}{\partial {\bar{x}}^{2}}=\frac{\partial \hat{c}\left(\bar{x},\bar{t}\right)}{\partial \bar{t}}$$

The boundary condition Equation (A7) becomes a homogenized version:

(A13) $$\hat{c}\left(0,\bar{t}\right)=0$$

and the Equation (A8) becomes

(A14) $${\left.\frac{\partial \hat{c}\left(\bar{x},\bar{t}\right)}{\partial \bar{x}}\right|}_{\bar{x}=1}=-\bar{\alpha }\hat{c}\left(1,\bar{t}\right)$$

The initial condition (A9) becomes

(A15) $$\hat{c}\left(x,0\right)=-{\bar{X}}_{0}$$

### Appendix A.5. Solving the Diffusion Equation with Separation of Variables

We use the common separation of variables technique [28] to solve this differential equation. We assume a solution of the following form:

(A16) $$\hat{c}\left(\bar{x},\bar{t}\right)=\hat{X}\left(\bar{x}\right)\hat{T}\left(\bar{t}\right)$$

Plugging this into Equation (A6), and introducing constant *k*, we obtain the following:

$$\left(\frac{1}{\hat{X}}\right)\frac{\partial^{2}\hat{X}}{\partial {\bar{x}}^{2}}=\left(\frac{1}{\hat{T}}\right)\frac{\partial \hat{T}}{\partial \bar{t}}=k$$

which results in two differential equations:

(A17) $${\hat{X}}^{\prime \prime }-k\hat{X}=0$$

and

(A18) $${\bar{T}}^{\prime }-k\bar{T}=0$$

### Appendix A.6. The Solution

The solution of Equations (A17) and (A18) is obtained by taking three cases for variable *k*, i.e., positive, negative, and zero.

#### Appendix A.6.1. Case 1: *k* = 0

We previously solved the steady-state solution in Appendix A.2. We will now just confirm that there is only a trivial solution in terms of $\hat{c}$; we can show that $\hat{c}\left(x,t\right)=0$

Equation (A17) is

$${\hat{X}}^{\prime \prime }=0$$

which has the solution $\hat{X}=Ax+B$ The boundary condition (A7) then yields

$$\hat{c}\left(0,\bar{t}\right)=\hat{X}\left(0\right)=0=A\left(0\right)+B=B$$

and boundary condition (A13) yields

$${\hat{X}}^{\prime }\left(1\right)=-\bar{\alpha }\hat{X}\left(1\right)$$

With $\hat{X}=Ax+B$ and ${\hat{X}}^{\prime }=A$, we obtain the following:

$$A=-\bar{\alpha }\left(A+B\right)=-\bar{\alpha }\left(A\right)$$

or

$$A(1+\bar{\alpha })=0$$

with α≥0, we obtain A=0.

As a result, k=0 results in a trivial solution since A=0 and B=0.

#### Appendix A.6.2. Case 2: *k* = *μ*^2^ > 0

The ODE (A17) becomes

$${\hat{X}}^{\prime \prime }-\mu^{2}\hat{X}=0$$

which has the solution of

$$\hat{X}=c_{1}e^{\mu \bar{x}}+c_{2}e^{-\mu \bar{x}}$$

and

$${\hat{X}}^{\prime }=c_{1}\mu e^{\mu \bar{x}}-c_{2}\mu e^{-\mu \bar{x}}$$

The boundary condition (A13) then yields

$$\hat{c}\left(0,\bar{t}\right)=\hat{X}\left(0\right)=0=c_{1}+c_{2}$$

and boundary condition (A14) then yields

$${\hat{X}}^{\prime }\left(1\right)=c_{1}\mu e^{\mu }-c_{2}\mu e^{-\mu }=-\bar{\alpha }\hat{X}\left(1\right)=-\bar{\alpha }\left(c_{1}e^{\mu }+c_{2}e^{-\mu }\right)$$

Plugging in $c_{2}=-c_{1}$ into the above, we obtain the following:

$$c_{1}\left(\mu \left(e^{\mu }+e^{-\mu }\right)+\bar{\alpha }\left(e^{\mu }-e^{\mu }\right)\right)=0$$

which has a trivial solution for $c_{1}=0$. The quantity in parentheses is positive since μ and α are positive.

Thus, $c_{1}=0$, $c_{2}=0$, and $k=\mu^{2}<0$.

#### Appendix A.6.3. Case 3: *k* = −*μ*^2^ < 0

From ${\hat{X}}^{\prime \prime }+\mu^{2}\hat{X}=0$, we obtain the following:

$$\hat{X}\left(x\right)=c_{1}\cos\left(\mu x\right)+c_{2}\sin\left(\mu x\right)$$

and, therefore,

$${\hat{X}}^{\prime }\left(x\right)=-c_{1}\mu \sin\left(\mu x\right)+c_{2}\mu \cos\left(\mu x\right)$$

With boundary condition (A13), we write

$$\hat{c}\left(0,\bar{t}\right)=0=c_{1}\cos\left(\mu \left(0\right)\right)+c_{2}\sin\left(\mu \left(0\right)\right)$$

to obtain the following:

(A19) $$c_{1}=0$$

and with boundary condition (A14), we write

$${\left.\frac{\partial \hat{c}\left(\bar{x},\bar{t}\right)}{\partial \bar{x}}\right|}_{\bar{x}=1}=-c_{1}\mu \sin\left(\mu \right)+c_{2}\mu \cos\left(\mu \right)=c_{2}\mu \cos\left(\mu \right)=-\bar{\alpha }\hat{c}\left(1,\bar{t}\right)=-\bar{\alpha }c_{2}\sin\left(\mu \right)$$

to obtain the following:

(A20) $$c_{2}\left(\mu \cos\left(\mu \right)+\bar{\alpha }\sin\left(\mu \right)\right)=0$$

Since we are not interested in trivial solutions where $c_{2}=0$, we must have

(A21) $$\tan\left(\mu \right)=-\frac{\mu }{\bar{\alpha }}$$

This equation has an infinite number of solutions [28], which we denote as $\mu_{n}$, where

(A22) $$\left(n-1/2\right)\pi <\mu_{n}<n\pi$$

For each integer n≥1, we have

$$X_{n}=\sin\left(\mu_{n}x\right)$$

The time solution corresponding to (A18) is

$${\bar{T}}^{\prime }+\mu^{2}\bar{T}=0$$

is

$$T_{n}=e^{-\mu_{n}^{2}t}$$

so that the normal modes of the solution are

$${\hat{c}}_{n}=X_{n}\left(x\right)T_{n}\left(t\right)=\sin\left(\mu_{n}x\right)e^{-\mu_{n}^{2}t}$$

The superposition of these modes provides the general solution:

$$\hat{c}\left(x,t\right)=\sum_{n=1}^{\infty }b_{n}\sin\left(\mu_{n}x\right)e^{-\mu_{n}^{2}t}$$

To compute coefficients $b_{n}$, we can use [28]:

(A23) $$b_{n}=\frac{\int_{0}^{1}f\left(x\right)\sin\left(\mu_{n}x\right)\;dx}{\int_{0}^{1}\sin^{2}\left(\mu_{n}x\right)\;dx}$$

where f(x), the initial value, is $f\left(x\right)=-{\hat{X}}_{0}=-\left(1-\frac{\bar{\alpha }x}{1+\bar{\alpha }}\right)=-1+\frac{\bar{\alpha }x}{1+\bar{\alpha }}$ This results in

$$b_{n}=\frac{-4\left(1+\bar{\alpha }\right)\mu_{n}+4\mu_{n}\cos\left(\mu_{n}\right)+4\bar{\alpha }\sin\left(\mu_{n}\right)}{\left(1+\bar{\alpha }\right)\mu_{n}\left(2\mu_{n}-\sin\left(2\mu_{n}\right)\right)}$$

To solve the equation for c(x,t), we perform the following steps:

- We calculate the coefficients $\mu_{n}$ using (A21) and (A22).
- We calculate the Fourier coefficients $b_{n}$ using (A23) up to the desired precision.
- We calculate $\hat{c}\left(\bar{x},\bar{t}\right)$.
- We calculate $\bar{c}\left(\bar{x},\bar{t}\right)$.
- We calculate c(x,t).

and $\bar{c}$ is

(A24) $$\bar{c}=\hat{c}+{\bar{X}}_{0}=\sum_{n=1}^{\infty }b_{n}\sin\left(\mu_{n}\bar{x}\right)e^{-\mu_{n}^{2}\bar{t}}+1-\frac{\bar{\alpha }\bar{x}}{1+\bar{\alpha }}$$

and $c_{Robin}$ is

(A25) $$c_{Robin}\left(x,t\right)=C_{0}\left(\sum_{n=1}^{\infty }b_{n}\sin\left(\frac{\mu_{n}x}{L}\right)e^{\frac{-\mu_{n}^{2}tD}{L^{2}}}+1-\frac{\frac{\alpha L}{D}\frac{x}{L}}{1+\frac{\alpha L}{D}}\right)$$

Since this type of sensor is a particular type of flux-type sensor, the sensor response (see Equation (A1)) is

(A26) $$m_{Robin}\left(t\right)={\left.D\frac{\partial c}{\partial x}\right|}_{x=L}=\alpha c\left(L,t\right)=\alpha C_{0}\left(\sum_{n=1}^{\infty }b_{n}\sin\left(\mu_{n}\right)e^{\frac{-\mu_{n}^{2}tD}{L^{2}}}+\frac{1}{1+\frac{\alpha L}{D}}\right)$$
